# EGFR fluorescence in situ hybridisation assay: guidelines for application to non-small-cell lung cancer

**DOI:** 10.1136/jcp.2009.066548

**Published:** 2009-10-20

**Authors:** M Varella-Garcia, J Diebold, D A Eberhard, K Geenen, A Hirschmann, M Kockx, I Nagelmeier, J Rüschoff, M Schmitt, S Arbogast, F Cappuzzo

**Affiliations:** 1 University of Colorado Cancer Center, Aurora, Colorado, USA; 2 Luzerner Kantonsspital, Luzern, Switzerland; 3 Independent Consultant, San Francisco, USA; 4 Histogenex, Antwerp, Belgium; 5 Targos GmbH, Kassel, Germany; 6 Roche, Penzberg, Germany; 7 Istituto Clinico Humanitas, Milano, Italy

## Abstract

There is a need for predictive biomarkers that identify non-small-cell lung cancer (NSCLC) patients most likely to respond to epidermal growth factor receptor (EGFR) tyrosine kinase inhibitor (TKI) treatment. There are numerous potential candidates, although none has been proven in prospective clinical trials. The EGFR gene copy number evaluated by fluorescence in situ hybridisation (FISH) has been highlighted as one of the most effective markers for sensitivity to EGFR TKIs in large phase III, randomised placebo-controlled trials and has been used in clinical settings to assist physicians in defining the therapeutic regimen. The EGFR FISH assay has technical challenges and it is critical that detailed guidelines are provided to help clinical laboratories in performing and interpreting the test. Excellent assay reproducibility and portability rates among laboratories are crucial to guarantee that accurate clinical decisions can be made for patients with NSCLC. This article discusses the consensus outcomes of a global workshop convened to discuss key technical issues and standardise reading strategies for the EGFR FISH assay of NSCLC tumour tissue.

Lung cancer remains the leading cause of cancer-related death worldwide.^1^ The prognosis for advanced non-small-cell lung cancer (NSCLC) is poor^2^ and global 5-year survival rates are low, ranging between only 10% and 15%.^3^ ^4^ Standard chemotherapy for NSCLC has reached a plateau in its therapeutic efficacy^5^ ^6^; however, novel targeted agents that act on molecules in signalling pathways have emerged as effective agents in treating NSCLC^7^ and they have provided renewed optimism for patients with advanced disease. The epidermal growth factor receptor (EGFR) is a tyrosine kinase (TK) receptor that is expressed in 40–80% of patients with NSCLC.^8^ ^9^ Due to the important role of EGFR in cellular growth and proliferation, it has been proposed as a target for NSCLC therapy^10^ and several EGFR inhibitors are being evaluated as treatment options for patients with advanced NSCLC.^11^

The EGFR TK inhibitors (TKIs, eg, erlotinib (Tarceva; OSI Pharmaceuticals, Inc, Melville, New York, USA; Genentech, Inc, South San Francisco, California, USA; and F Hoffmann-La Roche Ltd, Basel, Switzerland) and gefitinib (Iressa; AstraZeneca Pharmaceuticals, Wilmington, Delaware, USA)) are further along in clinical development for NSCLC treatment than other EGFR-targeted therapies. Erlotinib is currently the only approved EGFR TKI for advanced NSCLC therapy in the USA and European Union; the 2-month survival advantage observed with erlotinib compared with placebo in the pivotal phase III BR.21 trial led to its approval for the second-line/third-line therapy of patients with advanced disease.^12^ While gefitinib is approved for use in Japan, it was not approved by the US Food and Drug Administration for the treatment of recurrent NSCLC because the pivotal ISEL (Iressa Survival in Lung Cancer) trial failed to demonstrate a significant increase in the overall survival (OS) of patients treated with gefitinib compared with placebo in this indication.^13^ The impact of cetuximab (an anti-EGFR antibody; Erbitux, Imclone Systems Inc, New York, USA) on the treatment of NSCLC is not yet clear. In the large FLEX trial, the cetuximab plus cisplatin/vinorelbine arm demonstrated a significant survival advantage,^14^ whereas in the smaller BMS-099 trial a similar but not significant trend was found in the cetuximab plus carboplatin+taxane arm.^15^

Strategies for patient selection using molecular diagnostics have the potential to increase the efficacy of these molecular-targeted therapies and optimise response to treatment in patients with advanced NSCLC. Research efforts are ongoing to develop and validate laboratory tests for assessment of positive and negative predictive markers of treatment response and survival. Notably, no predictive marker of survival benefit with anti-EGFR treatment efficacy has been demonstrated prospectively, although validation studies toward this end are ongoing. EGFR protein expression assessed by immunohistochemistry, EGFR gene copy number by fluorescence in situ hybridisation (FISH), and mutations in the EGFR or other downstream genes have been under investigation as potential biomarkers that may predict sensitivity to anti-EGFR therapy. Two large, randomised clinical trials of EGFR TKI monotherapy in second-line/third-line NSCLC have been retrospectively analysed for biomarkers that may predict response and survival benefit to EGFR TKIs: BR.21^16^ ^17^ and ISEL.^18^ Data from both trials supported EGFR FISH status as a potential predictive marker of tumour response and patient survival to TKIs. Recently, a phase II trial in patients with advanced NSCLC also demonstrated that cetuximab plus chemotherapy improved progression-free survival (PFS) and OS in EGFR FISH-positive patients compared with those who were FISH-negative.^19^ These results suggest that an assay to determine EGFR FISH status may be applicable for selection of patients for anti-EGFR therapies, although prospective validation of these results is warranted before the use of the marker is implemented for patient management.

The study by Cappuzzo *et al*^20^ was the first to show that high EGFR copy number correlated significantly with improved survival in patients treated with gefitinib. NSCLC patients were classified into six groups according to the ascending copy number of the EGFR gene, and individuals with EGFR high polysomy or gene amplification (defined as EGFR FISH-positive) had a significantly higher response rate, and a significantly longer time-to-progression (TTP) and survival than patients with no EGFR gene gain (defined EGFR FISH-negative). EGFR FISH status has since been investigated retrospectively in numerous trials with conflicting impact on OS (table 1).

**Table 1:** EGFR FISH status and impact on survival in selected randomised NSCLC trials with EGFR TKIs

Study/author	Treatment arm	No. of patients with EGFR FISH result	No. of patients with EGFR FISH-positive status*	HR for survival in EGFR FISH-positive patients†	Cut-off values for EGFR FISH-positive status
BR.21/Tsao *et al*^16^,^17^	Erlotinib (150 mg) versus placebo	159	56 (45)	0.43 (0.004)	High degrees of polysomy or EGFR gene amplification using Colorado scoring criteria
ISEL/Hirsch *et al*^18^	Gefitinib (250 mg) versus placebo	370	114 (31)	0.61 (0.067)	High polysomy (⩾4 copies of EGFR in ⩾40% of cells) or EGFR gene amplification
INVITE/Crino *et al*^22^	Gefitinib (250 mg) versus vinorelbine	158	54 (34)	2.88	NR
INTEREST/Kim *et al*^24^,^25^	Gefitinib (250 mg) versus docetaxel	374	174 (47)	1.09 (0.52)	High polysomy (⩾4 copies of EGFR in ⩾40% of cells) or EGFR gene amplification
TRIBUTE/Hirsch *et al*^26^	Carboplatin/paclitaxel+erlotinib (150 mg) versus carboplatin/paclitaxel+placebo	245	100 (41)	1.52 (0.083)	High polysomy (⩾4 copies of EGFR in ⩾40% of cells) or EGFR gene amplification

*Values in parentheses are percentages; †values in parentheses are p values.

EGFR, epidermal growth factor receptor; FISH, fluorescence in situ hybridisation; HR, hazard ratio; NR, not reported in manuscript; NSCLC, non-small-cell lung cancer; TKI, tyrosine kinase inhibitor.

All studies used the LSI EGFR/CEP 7 probe set (Abbott Molecular, Des Plaines, Illinois, USA).

In the single-arm TRUST study with erlotinib, EGFR FISH-positive patients showed an improved OS,^21^ in contrast EGFR FISH-positive patients in the randomised studies INVITE (gefitinib monotherapy),^22^ INTEREST (gefitinib monotherapy)^23^ ^24^ ^25^ and TRIBUTE (erlotinib+chemotherapy)^26^ demonstrated no improved benefit in survival with EGFR TKI treatment. Notably, in the TRIBUTE study, among EGFR FISH-positive patients, the TTP was longer in patients who received erlotinib, despite the lack of OS benefit. The variability seen in the published clinical evidence for the predictive value of the EGFR FISH assay in NSCLC could result from differences between the studies (eg, different patient populations, different study designs and treatment and control arms (TKI monotherapy versus placebo or TKI+chemotherapy versus chemotherapy)), as well as differences in the methodologies used by the various study laboratories for performing and interpreting the assay. Additionally, there is no clear definition for the scoring of atypical signals and/or gene clusters in the Colorado scoring method and a lack of a precise method for calculation of the EGFR ratio; this could lead to a different interpretation of signals among different labs and across different studies. These high technical and interpretive complexities encountered when performing, reading and interpreting the FISH assay reinforce the need for standardised testing procedures. Some recommendations for the standardised application and interpretation of molecular assays in NSCLC have been suggested for the clinical trial setting.^27^ In addition, preliminary laboratory performance guidelines for assessment of the EGFR FISH assay in the stratification of patients with NSCLC have been reported.^28^ However, detailed guidelines for correctly performing and interpreting the EGFR FISH assay are needed to ensure the high reproducibility and portability between testing laboratories, as these are crucial for the successful clinical application of the assay.

## Consensus guidelines for analysis and interpretation of the EGFR FISH assay

These consensus guidelines are the result of a global workshop convened to discuss key technical issues and standardise reading strategies for the EGFR FISH assay of NSCLC tumour tissue. All laboratories should also comply with all guidelines required by the regulatory agencies in their countries.

### Sample preparation and storage guidelines

Pre-analytical variables can affect the final result of the EGFR FISH assay. For consistently robust and reproducible results, standardisation of the pre-analytical conditions and processes, as well as those of the assay itself, and interpretation of results are needed. Largely, the recommendations regarding pre-analytical variables are similar to the recommended guidelines for HER2 FISH testing in breast carcinoma.^29^

#### Fixation and storage of tumour specimens

The duration of fixation and the type of fixative used are key issues that could affect the viability of samples and modify subsequent FISH results. Samples should be fixed for greater than 6 h but no more than 48 h in 10% neutral buffered formalin/4% neutral buffered formaldehyde. The date and duration of fixation should be recorded for each specimen whenever feasible. Bouin’s, mercury-containing fixatives and any fixatives that would destroy DNA should be avoided. Ideally, tumour samples should be stored as blocks (formalin fixed, paraffin embedded) at room temperature, rather than as cut tissue sections.

#### Selection of tumour specimens

Tumour content of the specimen should be confirmed in a H&E-stained slide serial to the unstained sections prior to performing subsequent steps of FISH assays or prior to sending the sample to an external laboratory. It is preferable to use the diagnostic tumour blocks to ensure availability of a sufficient number of viable, morphologically intact tumour cells to fulfil the scoring requirements (see below) and histopathological representation of the patient’s tumour as a whole.

#### Sectioning of tissue

Positively charged slides should be used for mounting of tissue sections in order to avoid detachment of the tissue. The recommended thickness of tissue sections used for the EGFR FISH assay is 4 μM (range 1 μM), and the sectioning date should be recorded. If slides with sections are stored prior to testing, the date of sectioning and section storage conditions (room temperature, protected from light and humidity) should also be noted.

### EGFR FISH assay

Laboratories may follow the standard operating procedures that have proved to be successful for formalin-fixed, paraffin-embedded tissue sections in their settings for performing the assay. Attention must be paid to particular steps in the protocol that may affect probe penetration and hybridisation to target DNA in order to optimise signal intensities. The deparaffinisation process must remove all traces of residual paraffin, therefore the solvents should be changed for fresh solvents on a regular and frequent basis according to the number of sections being processed. Modifications in the protease digestion protocol may be required for specific cases (eg, large surgical specimens), and small biopsies may require different digestion times, as well as freshly cut and previously stored slides. Samples with abundant mucinous cells or dense fibrous tissue may require longer digestion times than those with non-mucinous or more delicate stromal matrix.

After hybridisation, sealing the coverslips in place with rubber cement, finger nail polish, etc, is not necessary; however large coverslips should be used to avoid mixing of immersion oil and the 4′,6-diamidino-2-phenylindole (DAPI)/anti-fade mounting medium. The hybridised slides should be stored in the dark at −20°C to +4°C until analyses and review are completed. Microscope analysis is recommended to be performed within 1 week of the assay in order to guarantee the optimal fluorescence intensity.

### Reading the EGFR FISH assay

#### Qualifications of personnel reading EGFR FISH

A properly trained reader who has undergone training in FISH should analyse the slides. The reader also should have had training on the pathological appearance of lung cancer, and should have easy access to assistance from an expert lung pathologist. The competency of all technical personnel reading and interpreting the results should be evaluated at least annually and continuous training should be ensured. The participation of the laboratory in quality assurance/quality control programmes is highly advised, as recommended in the HER2 testing guidelines.^29^

#### Quality assessment of the EGFR FISH specimen

**Figure 1:**
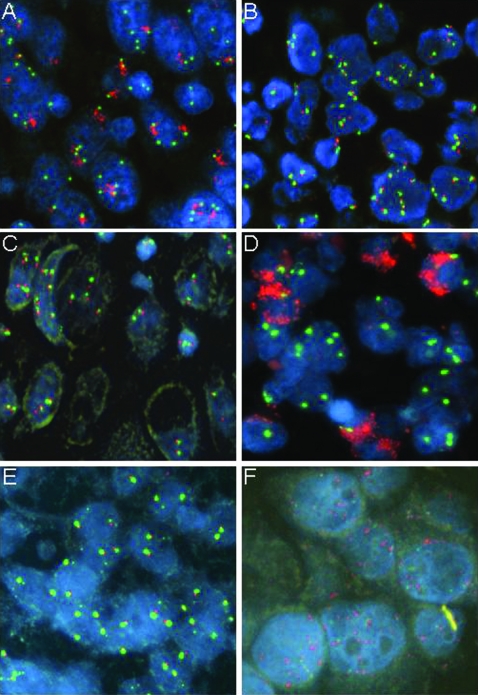
Quality assessment of non-small-cell lung cancer section subjected to the epidermal growth factor receptor fluorescence in situ hybridisation assay. (A, B) Specimens acceptable for analysis, showing well-defined nuclear morphology and excellent intensity of the fluorescent signals. (C–F) Specimens not acceptable for analysis because there are missing areas of nuclear chromatin (C), they show large autofluorescent structures obscuring the probe signals (D), they have poorly defined nuclear borders (E), or the intensity of the fluorescent signals is poor (F).

**Figure 2:**
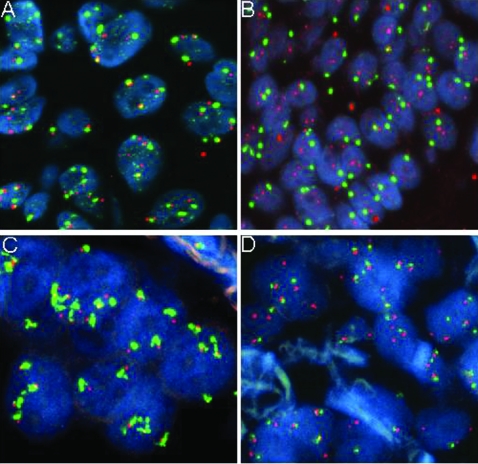
Inadequate signal quality in non-small-cell lung cancer sections for the epidermal growth factor receptor fluorescence in situ hybridisation assay that direct to subjective judgment. (A) Cross-hybridisation of the CEP7 probe with multiple unspecific targets generating multiple faint green signals. (B) Particulate background noise showing up as large red fluorescent spots. (C) Very stringy green signals. (D) Blotchy and patchy red signals, while the green signals are faint.

Once the assay control slides have been assessed and passed quality criteria, the quality of each tumour specimen must be assessed to determine its acceptability for analysis. At least 50% of tumour cells have to pass the assessment test fulfilling the following criteria prior to the actual reading and scoring. (a) Verification of integrity of the tumour nuclear morphology using the DAPI filter. Within adequate specimens, the chromatin of tumour cells should be well defined and non-disrupted (fig 1A, B). The preparation should not be undertreated to the point of preventing clear identification of the nuclear border, or overtreated with chromatin missing from nuclear areas. Tumour nuclei should not be covered by a cloudy yellowish layer or obscured by autofluorescent structures. Examples of specimens that do not pass quality assessment are illustrated in fig 1C–F. (b) Verification of the quality of the hybridisation in tumour and non-tumour cells using single-pass and double-pass interference filters. Green signals (CEP7) should be bright, compact (occasionally slightly stringy or diffuse) oval shapes, and red (EGFR) signals should be bright, small round shapes (fig 1A, B). The CEP7 signal is usually larger and brighter than the EGFR red signal and they are commonly adjacent. Signals should not be fuzzy, very patchy or blotchy. Representative images of those conditions are provided in fig 2A–D. (c) Confirmation that the background of the specimen appears dark and free of fluorescent particles or haziness under inspection with single-pass and double-pass interference filters.

#### Selection of tumour areas and tumour nuclei

Using H&E-guided microscopy prior to FISH reading, the histopathological features of the tumour should be assessed, including identification and characterisation of invasive tumour area(s), cell size, cell shape, differentiation, growth pattern, and presence of inflammatory or necrotic areas, which should be avoided. The tumour foci should always be identified on the H&E-stained slide by a lung pathologist, independently of whether the FISH analysis is to be performed by a pathologist or not.

Five areas representative of the histopathological features of the invasive part of the tumour must be selected. The selected areas are identified in the FISH slide with the DAPI filter, and within each area an average of 10 nuclei should be selected for analysis, for a total of 50 counted nuclei.

**Figure 3:**
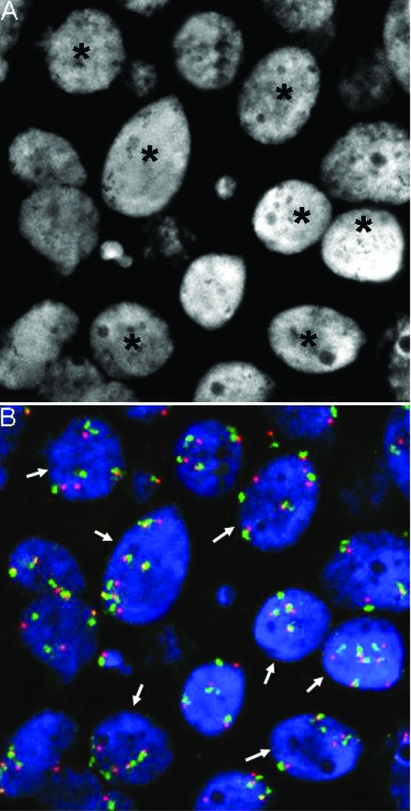
Selection of tumour cells to score. (A) High-magnification field under 4′,6-diamidino-2-phenylindole (DAPI) filter showing seven nuclei that qualify to be scored (indicated by asterisks) since they have average or larger size, non-disrupted chromatin, and intact and non-overlapping borders. (B) The same field with merged blue, red and green channels showing that all the selected nuclei (indicated by arrows) carry at least one signal for each probe. Therefore, all seven nuclei qualify for scoring.

Selection of tumour nuclei for reading should be performed using high-magnification objectives according to the following criteria: (a) with the DAPI filter, identify tumour nuclei of average size or larger, with non-disrupted chromatin and non-overlapping borders (as illustrated in fig 3); (b) with the single green, single red or dual red/green filters, confirm that selected cells carry at least one copy of each EGFR and CEP7 signal.

The areas and level of heterogeneity in the tissue should be noted, if heterogeneity is present. Tumours may be heterogeneous in their susceptibility to protease (eg, depending on extracellular matrix composition) and the quality of the EGFR signal may be different among these areas.

#### Enumeration of signals

**Figure 4:**
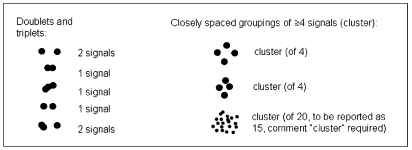
Guide for signal enumeration when epidermal growth factor receptor spots show up as doublets, triplets or clusters.

**Figure 5:**
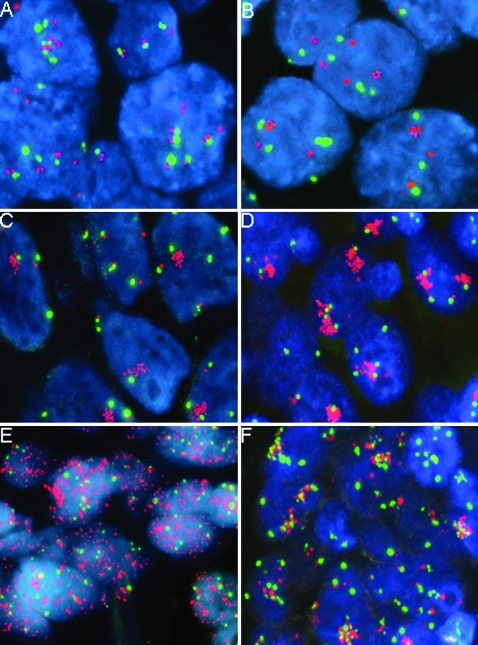
Examples of challenging scenarios for signal enumeration. (A) Nuclei with doublet and triplet spots counted as one signal each plus “paired” spots counted as two signals. (B–D) Nuclei with epidermal growth factor receptor (EGFR) gene amplification represented by clusters of signals probably due to HSR (homogeneously staining region). Clusters may include few spots (minimum of four spots, illustrated in B), intermediate (C) or very high numbers of spots (D). (E) Nuclei with EGFR gene amplification represented as numerous double minutes. (F) Nuclei with clusters of EGFR and CEP7 signals. In each nucleus, signals are enumerated as precisely as possible focusing up and down. If more than 15 signals are counted (which is a common finding in specimens with large clusters and double minutes) report “16” in the analysis worksheet.

The number of EGFR and CEP7 signals in the selected nuclei should be counted with single-pass interference filters (one filter for each probe wavelength) at ×60 to ×100 magnification and recorded separately. In the signal enumeration, there are special situations that require careful attention, such as when signals are split, paired, or in triplets or clusters (figs 4 and 5). Doublet or triplet spots that are physically linked, ie, touching or linked by a thread, or adjacent (with a gap smaller than the diameter of the largest signal), are counted as one signal only. However, spots that are adjacent but separated by at least the diameter of the largest signal are counted as separate signals. The maximum number of signals to be counted in a single cluster is 15. If signals are >15 the cluster is considered as innumerable and reported as “16”. A number is still required for calculation of the overall EGFR/CEP7 ratio, but counting in this range becomes increasingly difficult.

Due to the variety of mechanisms of EGFR amplification and clustering patterns noted in NSCLC samples, recommendation is provided in table 2 on how to score and interpret clusters of EGFR and CEP7 signals in these unique cases. Raw data should always be carefully recorded and stored, as they may be found to be useful in future studies.

**Table 2:** Updated Colorado score for scoring and interpreting clusters (adapted from Varella-Garcia, 2006^28^)

Pattern description	Scoring criteria	Expected ratio EGFR to CEP7 in the specimen	EGFR FISH classification*
Large clusters of EGFR signals	Count signals thoroughly and calculate the indexes. If number of signals is >15, write “16”	>2	Classify as positive (GA) if EGFR/CEP7 ratio ⩾2 or if clusters of EGFR signals are present in ⩾10% cells
Small clusters of EGFR signals (⩾4 signals)	Count signals thoroughly and calculate the indexes	>2 to ∼1 if high level of aneusomy 7 is present	Classify as positive (GA) if EGFR/CEP7 ratio ⩾2 or if clusters of EGFR signals are present in ⩾10% cells
Co-localised clusters of EGFR and CEP7 signals	Count signals thoroughly and calculate the indexes. If number of signals is >15, write “16”	∼1	Classify as positive (GA) if clusters are present in ⩾10% cells
Very high number of balanced red (EGFR) and green (CEP7) signals	Count signals thoroughly and calculate the indexes	∼1	Classify as positive (GA) if ⩾10% cells have ⩾15 EGFR signals
EGFR as double minutes	Count signals thoroughly and calculate the FISH indexes	>2	Classify as positive (GA) if EGFR/CEP7 ratio ⩾2 or ⩾10% cells have ⩾15 EGFR signals

EGFR, epidermal growth factor receptor; FISH, fluorescence in situ hybridisation; GA, EGFR gene amplification.

#### Number of readers for EGFR FISH

In its implementation at the University of Colorado (see Interpretation of the EGFR FISH results according to the Colorado scoring criteria), the EGFR FISH assay was scored by two independent readers, with a third reader involved when there was discordance between the first and second. Because of increased demand for efficiently testing large numbers of patients, a study was performed to establish a FISH evaluation procedure that would minimise the number of readers and reduce the number of specimens requiring multiple readers (L Morrison, personal communication 2008). The study utilised a mathematical approach to identify an equivocal range to determine if the sample was FISH positive or FISH negative. Based on the results of this study, the consensus recommendation was to set the high end of the equivocal range as 40% cells with ⩾4 signals, above which the first reader’s result classifies the specimen as EGFR FISH-positive. The low end was set at 25% of cells with ⩾4 signals, below which the first reader’s result classifies the specimen as EGFR FISH-negative. If the score of the first reader was within the equivocal or borderline range of >25% to <40% of cells with ⩾4 signals, then a second reader, blinded to the results of the first reader, was required. If the second reader reaches a positive score the specimen is concluded as FISH positive, and as FISH negative otherwise.

### Interpretation of the EGFR FISH results according to the Colorado scoring criteria

A scheme for classifying NSCLC tumours as EGFR FISH-positive and FISH-negative was developed at the University of Colorado and has been used in multiple clinical studies.^20^ ^28^

#### EGFR FISH-positive

(A) Specimens that have ⩾40% of cells displaying ⩾4 copies of the EGFR signal.

(B) Specimens that display EGFR gene amplification, defined according to one of the following criteria: (a) an EGFR to CEP7 ratio ⩾2 over all scored nuclei and calculated using the sum of EGFR divided by the sum of CEP7 when mean CEP7 per cell is ⩾2 copies; (b) the presence of gene cluster (⩾4 spots) in ⩾10% of tumour cells; (c) at least 15 copies of the EGFR signals in ⩾10% of tumour cells.

#### EGFR FISH-negative

Specimens that do not display gene amplification according to the criteria defined above and with <40% of cells displaying ⩾4 copies of the EGFR signal.

### Future directions and conclusions

There are several areas that need further investigation. For instance, the application of the Colorado scoring system requires adaptation and subsequent validation for cytological specimens and smears since those specimens include whole cells and do not have truncated nuclei as in the tissue sections.

The unique biology of advanced-stage NSCLC also pose difficulties associated with tumour heterogeneity that might get unaccounted for in small biopsies or even in very large specimens. Potential differences may exist in the genomic status of EGFR in pretreated versus untreated patients and the vast majority of information currently available was generated in diagnostic specimens from patients who had not received prior treatment. The predictive value of EGFR FISH in primary tumours compared with their corresponding metastases is another issue that requires confirmation in future clinical trials. Yet to be determined are the potential benefits or drawbacks of the FISH technology versus the related methods of chromogenic in situ hybridisation/silver in situ hybridisation for evaluation of the copy number of the EGFR gene in NSCLC.

Last, the development of robust quality assurance programmes and regular proficiency and competency assessment of laboratories performing these assays are necessary to improve the consistency of assay performance and interpretation. It is our intention that these guidelines will provide a tool to help the clinical laboratories involved with the EGFR FISH test in NSCLC to accomplish these goals.

**Take-home messages**

- Data from epidermal growth factor receptor (EGFR) fluorescence in situ hybridisation (FISH) assessment in clinical trials vary greatly. This may be due to different patient populations, different study designs, and variability in the methods used by different laboratories for reading and interpreting FISH.
- Excellent assay reproducibility is crucial to ensure that accurate clinical decisions are made for patients with non-small-cell lung cancer, therefore guidelines are critical for clinical laboratories performing and interpreting EGFR FISH assays.
- The guidelines summarised in this manuscript provide information on the inclusion/exclusion criteria, reading and scoring of slides, assessment of signal clusters and assessment of borderline cases.

**Interactive multiple choice questions**

This JCP best practice article has an accompanying set of multiple choice questions (MCQs). To access the questions, click on BMJ Learning: Take this module on BMJ Learning from the content box at the top right and bottom left of the online article. For more information please go to: http://jcp.bmj.com/education Please note: the MCQs are hosted on BMJ Learning—the best available learning website for medical professionals from the BMJ Group. If prompted, subscribers must sign into JCP with their journal’s username and password. All users must also complete a one-time registration on BMJ Learning and subsequently log in (with a BMJ Learning username and password) on every visit.

## References

[1] Parkin DM Bray F Ferlay J Global cancer statistics, 2002. CA Cancer J Clin 2005;55:74–108 15761078 10.3322/canjclin.55.2.74

[2] Molina JR Yang P Cassivi SD Non-small cell lung cancer: epidemiology, risk factors, treatment, and survivorship. Mayo Clin Proc 2008;83:584–94 18452692 10.4065/83.5.584 PMC2718421

[3] Sant M Aareleid T Berrino F EUROCARE-3: survival of cancer patients diagnosed 1990–94—results and commentary. Ann Oncol 2003;14:v61–118 14684501 10.1093/annonc/mdg754

[4] Ries L Melbert D Krapcho M SEER cancer statistics review, 1975–2005. Bethesda, MD: National Cancer Institute http://seer.cancer.gov/csr/1975_2005/ (accessed 7 July 2009)

[5] Schiller JH Harrington D Belani CP Comparison of four chemotherapy regimens for advanced non-small-cell lung cancer. N Engl J Med 2002;346:92–8 11784875 10.1056/NEJMoa011954

[6] Scagliotti GV De Marinis F Rinaldi M Phase III randomized trial comparing three platinum-based doublets in advanced non-small-cell lung cancer. J Clin Oncol 2002;20:4285–91 12409326 10.1200/JCO.2002.02.068

[7] Reck M Crino L Advances in anti-VEGF and anti-EGFR therapy for advanced non-small cell lung cancer. Lung Cancer 2009;63:1–9 18579254 10.1016/j.lungcan.2008.05.015

[8] Wheatley-Price P Shepherd FA Epidermal growth factor receptor inhibitors in the treatment of lung cancer: reality and hopes. Curr Opin Oncol 2008;20:162–75 18300766 10.1097/CCO.0b013e3282f335a3

[9] Sequist LV Lynch TJ EGFR tyrosine kinase inhibitors in lung cancer: an evolving story. Annu Rev Med 2008;59:429–42 17716025 10.1146/annurev.med.59.090506.202405

[10] Mendelsohn J Baselga J Epidermal growth factor receptor targeting in cancer. Semin Oncol 2006;33:369–85 16890793 10.1053/j.seminoncol.2006.04.003

[11] Ciardiello F Tortora G EGFR antagonists in cancer treatment. N Engl J Med 2008;358:1160–74 18337605 10.1056/NEJMra0707704

[12] Shepherd FA Rodrigues PJ Ciuleanu T Erlotinib in previously treated non-small-cell lung cancer. N Engl J Med 2005;353:123–32 16014882 10.1056/NEJMoa050753

[13] Thatcher N Chang A Parikh P Gefitinib plus best supportive care in previously treated patients with refractory advanced non-small-cell lung cancer: results from a randomised, placebo-controlled, multicentre study (Iressa Survival Evaluation in Lung Cancer). Lancet 2005;366:1527–37 16257339 10.1016/S0140-6736(05)67625-8

[14] Pirker R Szczesna A von Pawel J FLEX: A randomized, multicenter, phase III study of cetuximab in combination with cisplatin/vinorelbine (CV) versus CV alone in the first-line treatment of patients with advanced non-small cell lung cancer (NSCLC) [abstract]. J Clin Oncol 2008;26:6s

[15] Imclone Systems Incorporated Imclone press release: Erbitux(R) Phase 3 BMS-099 lung cancer study secondary endpoint update: overall survival results announced. http://phx.corporate-ir.net/phoenix.zhtml?c = 97689&p = irol-newsArticle&ID = 1192099&highlight = (accessed 7 July 2009)

[16] Tsao MS Sakurada A Cutz JC Erlotinib in lung cancer - molecular and clinical predictors of outcome. N Engl J Med 2005;353:133–44 16014883 10.1056/NEJMoa050736

[17] Zhu CQ da Cunha SG Ding K Role of KRAS and EGFR as biomarkers of response to erlotinib in National Cancer Institute of Canada Clinical Trials Group Study BR.21. J Clin Oncol 2008;26:4268–75 18626007 10.1200/JCO.2007.14.8924

[18] Hirsch FR Varella-Garcia M Bunn PA Jr Molecular predictors of outcome with gefitinib in a phase III placebo-controlled study in advanced non-small-cell lung cancer. J Clin Oncol 2006;24:5034–42 17075123 10.1200/JCO.2006.06.3958

[19] Hirsch FR Herbst RS Olsen C Increased EGFR gene copy number detected by fluorescent in situ hybridization predicts outcome in non-small-cell lung cancer patients treated with cetuximab and chemotherapy. J Clin Oncol 2008;26:3351–7 18612151 10.1200/JCO.2007.14.0111 PMC3368372

[20] Cappuzzo F Hirsch FR Rossi E Epidermal growth factor receptor gene and protein and gefitinib sensitivity in non-small-cell lung cancer. J Natl Cancer Inst 2005;97:643–55 15870435 10.1093/jnci/dji112

[21] Schneider C-P Heigener D Schott-von-Romer K EGFR-related tumor markers and clinical outcomes with Erlotinib in NSCLC: an analysis of patients from German centers in the TRUST Study. J Thorac Oncol 2008;12:1446–53 19057271 10.1097/JTO.0b013e31818ddcaa

[22] Crino L Cappuzzo F Zatloukal P Gefitinib versus vinorelbine in chemotherapy-naive elderly patients with advanced non-small-cell lung cancer (INVITE): a randomized, phase II study. J Clin Oncol 2008;26:4253–60 18779612 10.1200/JCO.2007.15.0672

[23] Douillard J Hirsh V Mok T Molecular and clinical subgroup analyses from a phase III trial comparing gefitinib with docetaxel in previously treated non-small cell lung cancer (INTEREST) [abstract]. J Clin Oncol 2008;26 (Suppl):no. 8001

[24] Douillard J-Y Kim ES Hirsh V Gefitinib (IRESSA) versus docetaxel in patients with locally advanced or metastatic non-small cell lung cancer pre-treated with platinum-based chemotherapy: a randomized, open-label Phase III study (INTEREST). J Thorac Oncol 2007;2:S305–6

[25] Kim ES Hirsh V Mok T Gefitinib versus docetaxel in previously treated non-small-cell lung cancer (INTEREST): a randomised phase III trial. Lancet 2008;372:1809–18 19027483 10.1016/S0140-6736(08)61758-4

[26] Hirsch FR Varella-Garcia M Dziadziuszko R Fluorescence in situ hybridization subgroup analysis of TRIBUTE, a phase III trial of erlotinib plus carboplatin and paclitaxel in non-small cell lung cancer. Clin Cancer Res 2008;14:6317–23 18829515 10.1158/1078-0432.CCR-08-0539 PMC3368373

[27] Eberhard DA Giaccone G Johnson BE Biomarkers of response to epidermal growth factor receptor inhibitors in Non-Small-Cell Lung Cancer Working Group: standardization for use in the clinical trial setting. J Clin Oncol 2008;26:983–94 18281673 10.1200/JCO.2007.12.9858

[28] Varella-Garcia M Stratification of non-small cell lung cancer patients for therapy with epidermal growth factor receptor inhibitors: the EGFR fluorescence in situ hybridization assay. Diagn Pathol 2006;1:19 16911776 10.1186/1746-1596-1-19 PMC1560164

[29] Wolff AC Hammond ME Schwartz JN American Society of Clinical Oncology/College of American Pathologists guideline recommendations for human epidermal growth factor receptor 2 testing in breast cancer. J Clin Oncol 2007;25:118–45 17159189 10.1200/JCO.2006.09.2775

